# Severe malaria intervention status in Nigeria: workshop meeting report

**DOI:** 10.1186/s12936-024-05001-1

**Published:** 2024-06-05

**Authors:** Emmanuel Shekarau, Miriam Uzoanya, Nnenna Ogbulafor, Severe Malaria Working Group, Godwin Ntadom, Simon Ntomchukwu Ijezie, Miriam Ihuoma Uzoanya, Babatunde Seye, Chizoba Fashanu, Nwamaka Eze, Lekia Nwidae, Olugbenga Mokuolu, Uchenna Nwokenna, Iniabasi Nglass, Olusesan Ishola-Gbenla, Methodius Okouzi, Motunrayo Fagbola, Olusola Oresanya, Dawit Getachew, Jennifer Chukwumerije, Victoria Erinle, Mohammed Kumo, Stephen Oguche, Jose Ambe, Hans Rietveld

**Affiliations:** National Malaria Elimination Programme, Public Health Department, Federal Ministry of Health, Abuja, Federal Capital Territory Nigeria

**Keywords:** Severe malaria, Pre-referral intervention, Guidelines, Referral system, Nigeria

## Abstract

Nigeria accounts for 39% of global malaria deaths in children under 5 years of age and the effective management of severe malaria is a health priority. The Annual Nigeria Severe Malaria Stakeholders Workshop, held on the 5–6th of July 2023 in Abuja, Nigeria brought together representatives from 36 States, the Federal Capital Territory, and other key stakeholders to address the management of severe malaria across all levels of the health service. Aims were to provide updates and review progress on severe malaria activities, the burden of disease, commodity logistics management, and pre-referral national policy implementation as well as to disseminate research findings. Two roundtable discussions were conducted to identify the challenges, barriers, and facilitators to the effective management of severe malaria in Nigeria. A key challenge was the limited awareness of updated guidelines and strategic documents among frontline health workers, leading to the misuse of non-recommended medications, like α-β-arteether. Further to this, the need to ensure appropriate treatments during pregnancy and the adoption of the WHO directive on the use of rectal artesunate were highlighted. To address these issues, innovative dissemination channels for guideline awareness were recommended and collaboration with professional organizations to enrich training materials emphasized. Other areas for improvement considered the processes involved in severe malaria management, with insufficient coordination among government agencies, inadequate referral linkages, and inadequate human resources identified as barriers. Recommendations focused on practical measures to minimize wastage of injectable artesunate, enhance data management through scaling up electronic medical records, and strengthen referral systems. The extension of severe malaria surveillance to patients older than 5 years was also proposed. To deliver these changes, actionable plans for sustained recruitment and training are needed, as well as committed advocacy at all levels to ensure timely fund disbursement and institutional support. A key overarching theme from the workshop was that a multifaceted approach was needed to address severe malaria in Nigeria, emphasizing collaborative efforts, evidence-based practices, and strategic resource allocation. With the largest malaria burden globally, the potential impact of addressing the challenges of severe malaria management in Nigeria cannot be understated and must be urgently addressed.

**Supplementary Information:**

The online version contains supplementary material available at 10.1186/s12936-024-05001-1.

## Background

The World Health Organization (WHO) African region accounted for 94% of malaria cases and 96% of deaths in 2022 [1]. Nigeria has the largest malaria burden in Africa and globally, with an estimated 66.7 million malaria cases in 2022, representing 27% of global malaria cases and 31% of deaths [1]. The severe form of malaria caused 189,321 deaths in Nigeria in 2022, with approximately 80% occurring in children under 5 years of age, accounting for 39% of global malaria deaths in this age group [1]. Thus, addressing severe malaria, particularly in young children, is a health priority for the country.

**Fig. 1:**
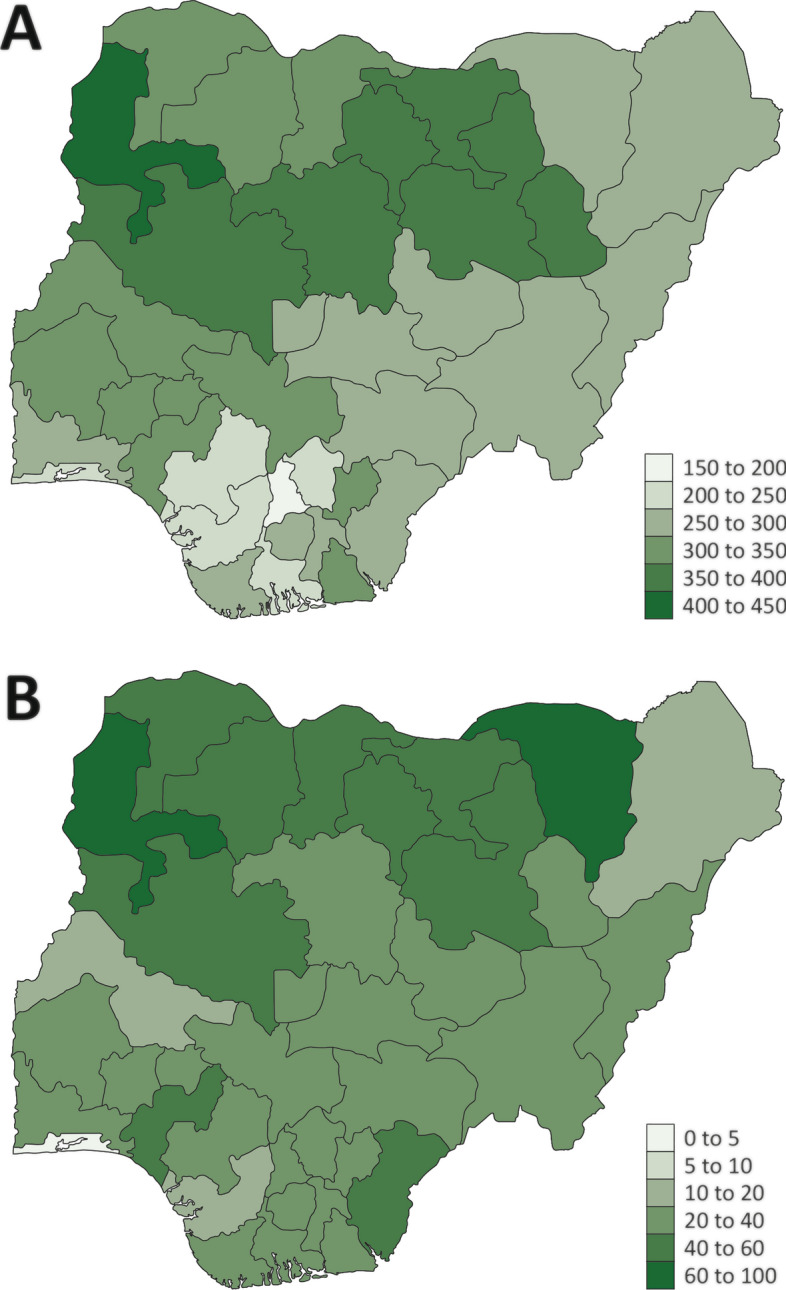
Burden of malaria in Nigeria in 2021 [3]. **A** Estimated malaria incidence per 1000 population. **B** Malaria prevalence (%) according to rapid diagnostic testing in children under 5 years of age [10]. Figure adapted from: Report on malaria in Nigeria 2022. Brazzaville: WHO Regional Office for Africa; 2023. Licence: CC BY-NC-SA 3.0 IGO

Malaria is endemic in Nigeria, with the entire population at risk. Transmission varies seasonally in the north and is more uniform throughout the year in the central and southern parts of the country [2]. An in-depth overview of the malaria situation across Nigeria, including demographics, malaria interventions, climate, and disease burden has been recently published [3]. Figure 1 summarizes the burden of disease across the country.

Over the last decade, the Government of Nigeria and its partners have implemented malaria prevention activities. By 2021, long-lasting insecticidal nets had been distributed to 37 states with around 41% of children protected, and the scaling-up of seasonal malaria chemoprevention reached 24 million children across 18 states [3–6]. Although the uptake of intermittent preventive treatment of malaria during pregnancy (IPTp) has been limited, a recent pilot study demonstrated the potential of community IPTp delivery [7, 8]. Malaria case management has been strengthened with the release of updated guidelines in 2020, requiring parasitological confirmation of all suspected malaria cases using microscopy or a rapid diagnostic test, and treatment with artemisinin-based combination therapy (ACT) [9]. The management of uncomplicated malaria has also been extended into the community to promote early diagnosis and prompt treatment, implemented through the establishment of the Community Health Influencers and Promoters of Services (CHIPS) network.

These interventions have generated improvements in malaria key indicators, with a 26% decline in malaria incidence and a 55% decrease in mortality between 2000 and 2021 [3]. Importantly, there has also been an overall decline in parasite prevalence in children under 5 years from 42% in 2010 to 22% in 2021 [10]. However, there is scope to further reduce the malaria burden by expanding coverage of preventive interventions. Perennial malaria chemoprevention (PMC) (formally intermittent preventive treatment of infants) is not currently deployed and malaria vaccination has not been implemented in Nigeria, with awareness of the vaccine in caregivers currently low [11]. Importantly, malaria mortality is still unacceptably high, underscoring the urgent need for comprehensive and effective strategies to effectively manage severe malaria cases [1].

The national response to the malaria situation in Nigeria is led by the National Malaria Elimination Programme (NMEP), an agency of the Federal Ministry of Health. The NMEP coordinates all malaria-related interventions, including severe malaria, at all three tiers of health management and service delivery levels (Federal, State and Local Government Areas [LGAs]), in concert with a robust partnership of non-governmental organizations, and other ministries and agencies of the government. A well-structured partnership coordination framework is operated under a National Technical Working Group, supported by subcommittees which are assigned by malaria intervention or ‘thematic’ areas. The Technical Working Group subcommittees, in turn, have ad hoc task teams based on specific service delivery areas under each intervention/thematic area. This coordination structure at the national level is also replicated in the 36 states plus the Federal Capital Territory (FCT) in Nigeria, though with varying degrees of functionality. Statutory meetings of the subcommittees and their subgroups are conducted monthly and quarterly, respectively, to review the status of implementation of thematic activities, troubleshooting challenges and constraints, resolving bottlenecks, and identifying solutions.

Nigeria is currently implementing the fifth National Malaria Strategic Plan 2021–2025. This sets ambitious goals to reduce the parasite prevalence to less than 10% and reduce mortality in children under 5 years of age to less than 50 deaths per 1000 live births by 2025 [2]. To put this in context, in 2018, the under-5 mortality rate was 132/1000 live births nationally and was as high as 252 deaths/1000 live births in Ogun State [2]. Reducing the malaria burden requires effective scale-up and strategic deployment of malaria interventions throughout the country. Epidemiological surveillance of cases and deaths attributable to malaria is required to provide information to accelerate the transition from control to elimination, and thereafter interruption of transmission. Availability of accurate and consistent information on malaria indices will facilitate early identification of populations at risk and identify foci and outbreaks. Prompt diagnosis and treatment using the recommended artemisinin-based combination therapy (ACT) according to the national treatment guidelines is critical. In addition, periodic monitoring of the therapeutic efficacy of anti-malarial medicines will allow for effective programmatic planning and timely responses and/or intensification of control measures. Thus, surveillance activities are crucial to inform targeted interventions in line with the National Strategic Plan.

Nigeria is one of 11 countries supported under the WHO high burden, high impact approach. This emphasizes political will and commitment from national to community level, strategic use of information for action, better technical and policy guidance at all levels, and effective coordination and leadership [1]. A key component of this approach is engagement with stakeholders to ensure they are up-to-date with epidemiological data, recent tools and innovations, ongoing malaria-related research initiatives and breakthroughs, partnerships and collaborations, as well as the specific challenges and barriers of severe malaria in Nigeria [2].

### Management of severe malaria in Nigeria

Severe malaria is a medical emergency requiring rapid clinical assessment and confirmation of the diagnosis followed by immediate treatment. The Nigerian malaria treatment guidelines recommend intravenous artesunate as the first-line treatment of severe *Plasmodium falciparum* malaria, though intramuscular artesunate can also be given [9]. If parenteral artesunate is not available, intramuscular artemether can be administered, but not α-β-arteether [9]. Within the community, pre-referral treatment can include (in order of preference), for children a single dose of rectal artesunate, or intramuscular artesunate, artemether, or quinine; and for adults intramuscular artesunate, artemether or quinine [9].

Severe malaria is the responsibility of the case management subcommittee, though specifically managed by the subcommittee’s Severe Malaria Working Group (SMWG). Members of the SMWG number around 50 and comprise representatives of the NMEP, development partners, such as WHO, the United Nations Children's Fund (UNICEF), and the United States Agency for International Development (USAID) Integrated Health Programme (IHP), as well as implementing non-governmental organizations such as Catholic Relief Services (CRS), Clinton Health Access Initiative (CHAI), Management Sciences for Health (MSH), and Malaria Consortium (MC). At its second quarterly meeting held on 10th May 2023, the SMWG reached a consensus to convene the 2023 Annual Severe Malaria Stakeholders Meeting.

The main purpose of the Annual Severe Malaria Stakeholders Meeting is to foster a consultative and engagement forum for a broad base of stakeholders, drawn from national and subnational levels. The aim is to provide updates on the status of programme implementation, identify future actions for improving service provision, and consolidate support for strengthening the delivery systems for the management of severe malaria in the country. Importantly, the meeting does not consider malaria prevention, but is focused on improving the management of severe malaria with the objective of improving patient outcomes. This paper summarizes the proceedings and outcomes of the 2023 Annual Severe Malaria Stakeholders Meeting held on 5th and 6th July 2023 in Abuja, Nigeria.

### Workshop objectives

The workshop considered the spectrum of issues relating to the management of severe malaria. Specific objectives of the meeting were to:

1. Provide an update and review of progress on severe malaria activities in the country (NMEP and partners’ projects), including the burden of the disease and commodity logistics management.
2. Disseminate recent research findings on severe malaria conducted globally and in-country and discuss implications for policy and implementation in Nigeria.
3. Provide an update and discuss ongoing efforts for routine data retrieval on severe malaria and ways to institutionalize this.
4. Define clear solutions and roadmaps to identify challenges with severe malaria management.

For the meeting agenda see Additional file 1. The meeting structure was a combination of plenary presentations with questions and answers and roundtable discussions in syndicate groups that employed a modified nominal group technique (without voting) to achieve consensus [12]. The workshop was formally opened by the Director of Public Health, Federal Ministry of Health, Nigeria, represented by the NMEP National Coordinator, Dr Perpetua Uhomoibhi. Opening remarks were followed by goodwill messages from partners and representatives of the Nigerian Medical Association and National Association of Nigeria Nurses and Midwives. The meeting was chaired by Professor Olugbenga Mokuolu, Portfolio Director of MSH.

### Participants

In addition to the National Coordinator of the NMEP, Heads of Branches and key technical staff, participants comprised Directors of hospital/medical services and paediatricians from tertiary hospitals in 31 States, Directors from Federal tertiary hospitals, and representatives from academia and professional associations (Nigerian Medical Association and National Association of Nigeria Nurses and Midwives, and the Paediatric Association of Nigeria). Partners’ representatives attended from the WHO, UNICEF, US President’s Malaria Initiative for States (PMI-S), Medicines for Malaria Venture (MMV), CRS, USAID IHP, MSH, MC, and CHAI.

## Day 1 presentations

### National guidelines for diagnosis and treatment

**Fig. 2:**
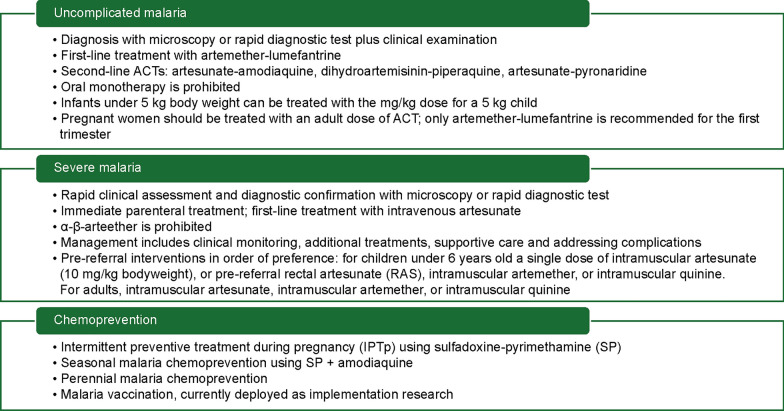
Summary of National Guidelines for Diagnosis and Treatment of Malaria in Nigeria (fourth edition, 2020) [9]

The fourth edition of the National Guidelines for Diagnosis and Treatment of Malaria in Nigeria, updated in 2020, was aligned with the National Malaria Strategic Plan [2], and the WHO guidelines for malaria [13]. It integrates new data and tools, including findings from therapeutic efficacy studies [14], to address changes in disease burden and demographics. Dr Emmanuel Shekarau from NMEP, Nigeria, outlined the recommendations for diagnosis and treatment of uncomplicated and severe malaria, and chemoprevention (Fig. 2). Treatment of uncomplicated malaria aims to promptly cure the infection and prevent progression to a severe form, whereas severe malaria is a medical emergency with treatment aiming to prevent death, disability, and recrudescence. Community management has been expanded to promote early diagnosis and prompt treatment with oral artemisinin-based combinations for uncomplicated malaria and parenteral artemisinin or pre-referral rectal artesunate (RAS) for severe malaria. Challenges to implementing the national malaria diagnosis and treatment guidelines include non-adherence, especially in the private sector, insufficient capacity and skills among some health workers, such as laboratory scientists, and inadequate equipment and facilities for case management.

### Community health influencers and promoters of services (CHIPS)

**Fig. 3:**
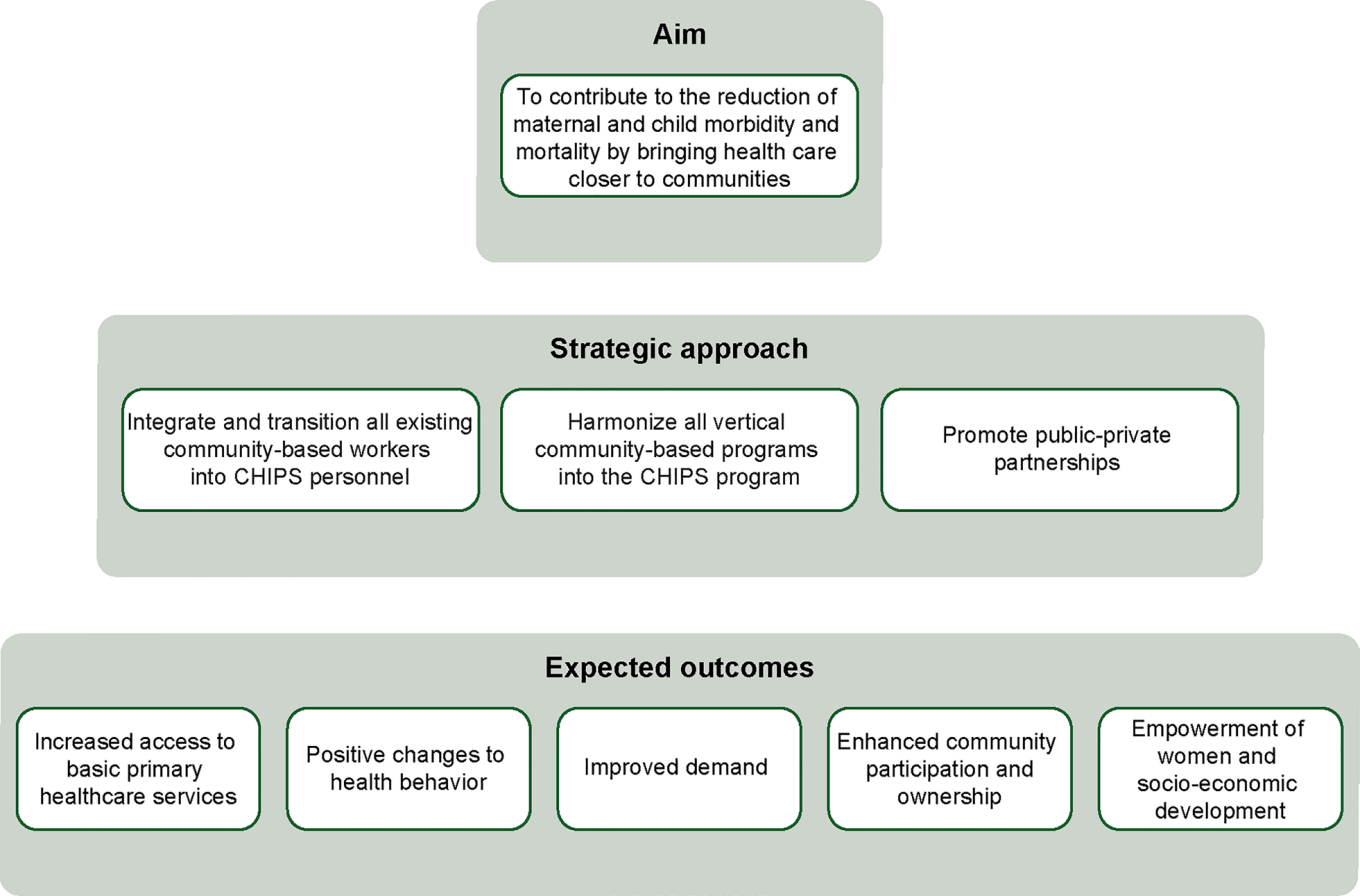
Overview of the Community Health Influencers, Promoters and Services (CHIPS) Programme

The CHIPS programme, a collaborative effort by the Federal Ministry of Health, National Primary Health Care Development Agency, and partners, is designed to strengthen the community component of primary healthcare (PHC) in Nigeria by harmonizing all community-based health programmes into a single national programme. Launched in 2018, CHIPS aims to reduce maternal and child morbidity and mortality by expanding access to primary care services (Fig. 3). CHIPS Agents are trained community members, primarily women, who deliver basic healthcare services through home visits. They are skilled communicators and provide preventive care, referrals, and health education and collect community data. About 10 are required per ward. Community Engagement Focal Persons (CEFPs) are typically male, aged 25 and above, fluent in the local language. Two per ward, they support CHIPS Agents by promoting male participation and by collecting and managing community data.

The CHIPS programme plays a pivotal role in identifying severe malaria in the community and promoting rapid treatment. CHIPS Agents assess danger signs for severe malaria using the ‘sick child form’. If severe malaria is suspected, they initiate pre-referral treatment with immediate referral to a health facility. They are also responsible for data collection. CEFPs are supported to verify and summarize data at the community level before transmission to the ward, LGA and State levels. The LGA level is responsible for validation to enhance data generation and flow and for the overall quality of reporting. Once validated, data are used to inform effective programme planning, monitoring and evaluation. By March 2023, CHIPS was implemented in 154 LGAs across 15 states and from January to March 2023, 25,801 confirmed malaria cases in children were treated with ACT in the community. By providing access to prompt malaria diagnosis and treatment in the community as well as improved data reporting and monitoring it is expected that fewer malaria cases will progress to severe malaria.

### Severe malaria retrospective studies

Severe malaria is a brief but serious illness needing urgent treatment. Treatment mainly happens in secondary and tertiary healthcare facilities. Although most malaria deaths occur in these facilities, cases are often not properly recorded in national statistics, making it hard to measure the effectiveness of interventions on mortality. Professor Stephen Oguche from Jos University Teaching Hospital, Plateau State, Nigeria, presented a retrospective analysis of severe malaria cases from July 2015 to June 2019 in tertiary and secondary healthcare facilities across Nigeria's six geopolitical zones.

Hospital records, including outpatient and inpatient malaria morbidity and mortality data, consultations and admissions, and interviews with healthcare workers, were examined for the study. Across 18 tertiary healthcare facilities, data were collected for 5159 patients with severe malaria. Nearly half of the cases were children < 5 years of age, but 40% were in children aged 5–15 years, and 10% were in those > 15 years old. In 17 secondary healthcare facilities, 3503 cases were reviewed, with the highest number in the north-central zone (37.9%) and lowest in the south-south (3.0%), with most cases (57%) occurring in children < 5 years of age, while 43% were in older children. Severe malaria cases were found across all social classes and over-diagnosis was more likely than under-diagnosis. Common complications were prostration, severe malarial anaemia, hypoglycaemia, hyperparasitaemia, and multiple convulsions, with sepsis and diarrhoea the most common co-morbidities. Mortality was 6%, and 40% of patients needed blood transfusion, which was a significant challenge at the secondary level of the health system because of the risk of blood-borne pathogens. These findings suggest that urgent attention is directed towards strengthening the secondary healthcare delivery system, including human resource capacity building and facility improvement, routine data collection and analysis, and the dissemination of case management information to healthcare workers. Additionally, continuous surveillance of case management practices and data reporting habits among healthcare workers was suggested to enhance routine reporting and improve malaria management.

### Death audit: findings from post-mortem investigation

A death audit reviews trends and causes of death related to severe malaria. It aims to identify factors that can be changed to prevent future deaths, implement those changes, and assess their effectiveness. It can also highlight systemic issues that need addressing to reduce severe malaria-related deaths [15].

Professor JP Ambe from the University of Maiduguri Teaching Hospital, Maiduguri Borno State in Nigeria outlined a study conducted in the South-West region of Nigeria (Ibadan) examining admissions in a tertiary hospital. Severe malaria accounted for 11.3% of all admissions, with 89.1% of cases occurring in children under 5 years old. Severe malaria accounted for 12.4% of all paediatric deaths, resulting in an estimated overall case fatality rate of 9.6%. Deaths from severe malaria were significantly associated with wasting, age under 2 years, hypoglycaemia, and respiratory distress. Other studies have associated malaria deaths with cerebral malaria, respiratory acidosis, renal failure and hypoglycaemia [16–19]. Co-morbidities, particularly sepsis, are often associated with severe malaria and require appropriate management [20, 21]. Contributing factors to malaria death include delayed patient presentation, insufficient assessment and documentation of cases, lack of supportive investigations, inadequate care of patients with reduced consciousness or shock, lack of access to renal replacement therapy or blood transfusion, and poor follow-up of patient progress [15, 22–26].

To inform improvements, the tools for death auditing should examine the quality of care relative to guidelines, capturing information on the number of staff trained in severe malaria management, procedures for artesunate injection preparation and usage, RAS usage, the triage system method and waiting time, the referral system and pre-referral treatment, and the availability of supporting equipment, as well as other structural issues that could contribute to late presentation or suboptimal management. Regular malaria death audit meetings are a potentially useful learning tool to improve patient care and reduce deaths from severe malaria.

### Improving the quality of care in Kano State

Lekia Nwidae from CHAI outlined a pilot project supported by CHAI, Nigeria to assist the Kano State Malaria Elimination Programme (SMEP) and State Primary Health Care Development Agency. This project aimed to enhance the quality of care by improving data quality and using data to strengthen malaria case management and adherence to treatment guidelines. The pilot involved 45 health facilities across three LGAs: Dawakin Tofa and Madobi as pilot LGAs, with Kano Municipal as the control LGA.

Baseline assessment in intervention and control LGAs showed deficiencies in treatment guidelines, poor adherence to malaria protocols, limitations in data quality and concordance, and inactive quality improvement teams at the LGA and PHC levels, leading to inadequate supervision of healthcare workers. The pilot aligned with Kano State’s Maternal Newborn Reproductive Child Health strategy, focusing on governance, service delivery, and data management. In intervention LGAs, governance was strengthened by integrating malaria quality of care into the state’s strategy and activating quality improvement teams. Service delivery was improved through capacity building for case management and data management, peer-led learning, and guideline provision. These interventions led to increased adherence to treatment guidelines; notably 85% of positive malaria cases were treated with an artemisinin-based combination compared to 71% at baseline. Automated dashboards strengthened data management, improving data quality and concordance. These data were applied to monitor stock consumption, guideline adherence, and to plan and monitor quality of care improvement training and mentoring interventions. The intervention significantly improved data quality in intervention LGAs, e.g., from 9% at baseline to 86% at the evaluation point in Dawakin Tofa. In summary, the pilot demonstrated that a comprehensive, collaborative, data-driven approach can enhance malaria quality of care. However, other issues such as the availability of resources, healthcare infrastructure, socioeconomic conditions, access to care, and regional disparities must also be considered. This requires a comprehensive approach involving collaboration between the government, all levels of the health system, communities, and partner organizations.

### PMI support for severe malaria intervention in Nigeria

Dr Augustine Firima from PMI-S, Nigeria, described how the PMI-S project, a 5-year initiative funded by USAID, supports the NMEP in combating malaria. It operates through task orders via the SMEP across eight states (Akwa Ibom, Benue, Cross River, Ebonyi, Nasarawa, Oyo, Plateau, and Zamfara). Its objectives are to enhance malaria case management and drug-based prevention strategies, improve data management, and strengthen health systems for malaria elimination in Nigeria.

For severe malaria, the project includes 226 secondary and 14 tertiary health facilities. Activities include strengthening malaria case management coordination systems, improving malaria diagnosis, supplying necessary equipment, providing guidelines, standard operating procedures and job aids, distributing malaria commodities, and training healthcare workers on severe malaria case management, laboratory scientists on malaria microscopy, and health records officers on data management. Challenges encountered include under-reporting and non-reporting of severe malaria cases, shortages and high costs of injectable artesunate, and the use of non-recommended drugs, such as α-β-arteether. Strategies to address these challenges were implemented through stakeholder engagement, with additional training and support for healthcare workers on the signs of severe malaria, sourcing of injectable artesunate, and capacity building for health workers on the use of recommended medications and severe malaria management.

### Malaria threats

A presentation by the WHO outlined the threats to malaria control. Genetic mutations in *P. falciparum* can lead to false-negative results in rapid diagnostic tests, impacting patient safety and transmission control. Monitoring for these mutations is crucial to adjust diagnostic and treatment strategies accordingly. Additionally, drug resistance is a persistent obstacle, and must be monitored through therapeutic efficacy studies, including molecular marker surveillance. Where resistance impacts clinical efficacy, prompt changes in treatment protocols are required to ensure patient outcomes. Surveillance of insecticide resistance in malaria vectors is vital for effective vector control, particularly as resistance is widespread and intensifying. Entomological surveillance is needed to combat the threat of invasive vector species, for example *Anopheles stephensi*, which may have behavioural or molecular adaptations that enable them to evade vector control. For all these potential threats, enhanced monitoring efforts are needed globally, with a focus on regions lacking sufficient data. Collaborative efforts and timely reporting of lessons learned are recommended to inform evidence-based strategies for malaria control and eradication.

### Automated software in the identification of malaria retinopathy

**Fig. 4:**
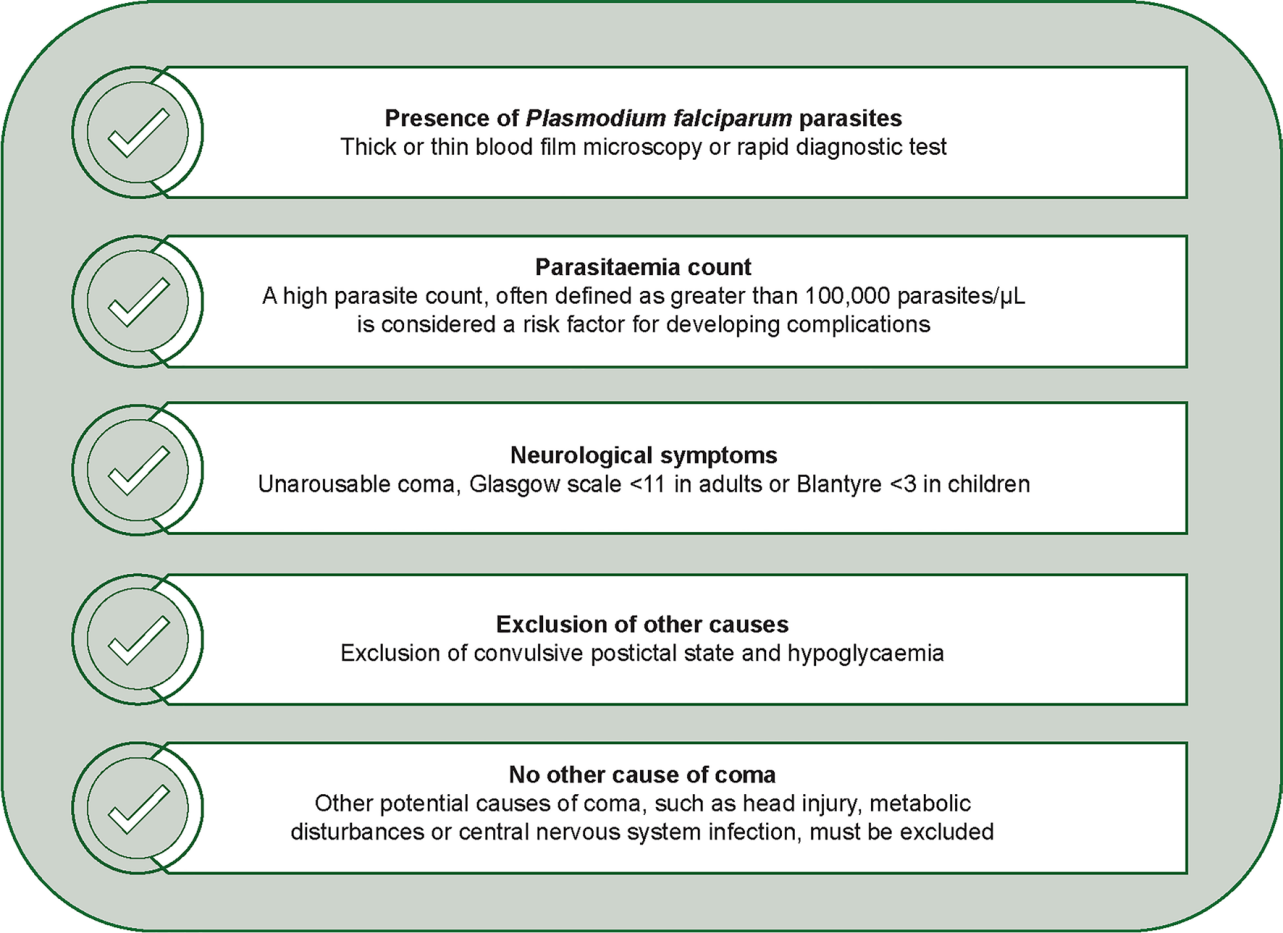
General criteria for the diagnosis of cerebral malaria [27]

Dr Folajimi Adebowale, AEyeCARE Biomedical Ltd described the potential utility of the *Automated Software in the Identification of Malaria Retinopathy in Digital Retina Images of Cerebral Malaria* – ASPIRE device for improving the diagnosis of cerebral malaria. General diagnostic criteria for cerebral malaria can result in misdiagnosis because parasitaemia can be incidental to coma in malaria endemic regions (Fig. 4) [27]. The retinal microvasculature represents an opportunity to visualize the effect of the parasitic invasion of neural tissue in vivo [28]. Malarial retinopathy correlates with mortality and the duration of coma in African children with cerebral malaria and improves the sensitivity and specificity of diagnosis [29–34]. However, retinal examination is not always feasible because of the set-up cost [35].

ASPIRE is a non-invasive handheld device designed to allow any healthcare worker to conduct retinal examinations on patients and autodetect malaria retinopathy in less than a minute. It was trained on > 100,000 retinal images, clinically validated in > 3000 patients, and field-tested in 26 clinics in Nigeria, Kenya, Ghana, and Malawi. Regulatory approval from the United States Food and Drug Administration and African countries has been applied for. ASPIRE can be used as a diagnostic, prognostic, and epidemiological tool to potentially enhance the quality of care for severe malaria and particularly cerebral malaria.

### Appropriate dosing of injectable artesunate

Following referral, injectable artesunate is the first-line treatment for severe malaria, though intramuscular artemether or intravenous quinine can be given if injectable artesunate is not available. The superiority of injectable artesunate over quinine for severe malaria is supported by two large-scale randomized studies. The African Quinine Artesunate Malaria Trial (AQUAMAT), which included sites in Nigeria, and the Southeast Asian Quinine Artesunate Malaria Trial (SEAQUAMAT), showed mortality risk was reduced with artesunate versus quinine by 22.5% and 34.7%, respectively [36, 37]. Both studies used an injectable artesunate dose of 2.4 mg/kg at 0, 12 and 24 h and then every 24 h until the patient could tolerate oral medication. Following the positive findings of AQUAMAT, Nigeria was the first country to adopt injectable artesunate for the management of severe malaria.

**Fig. 5:**
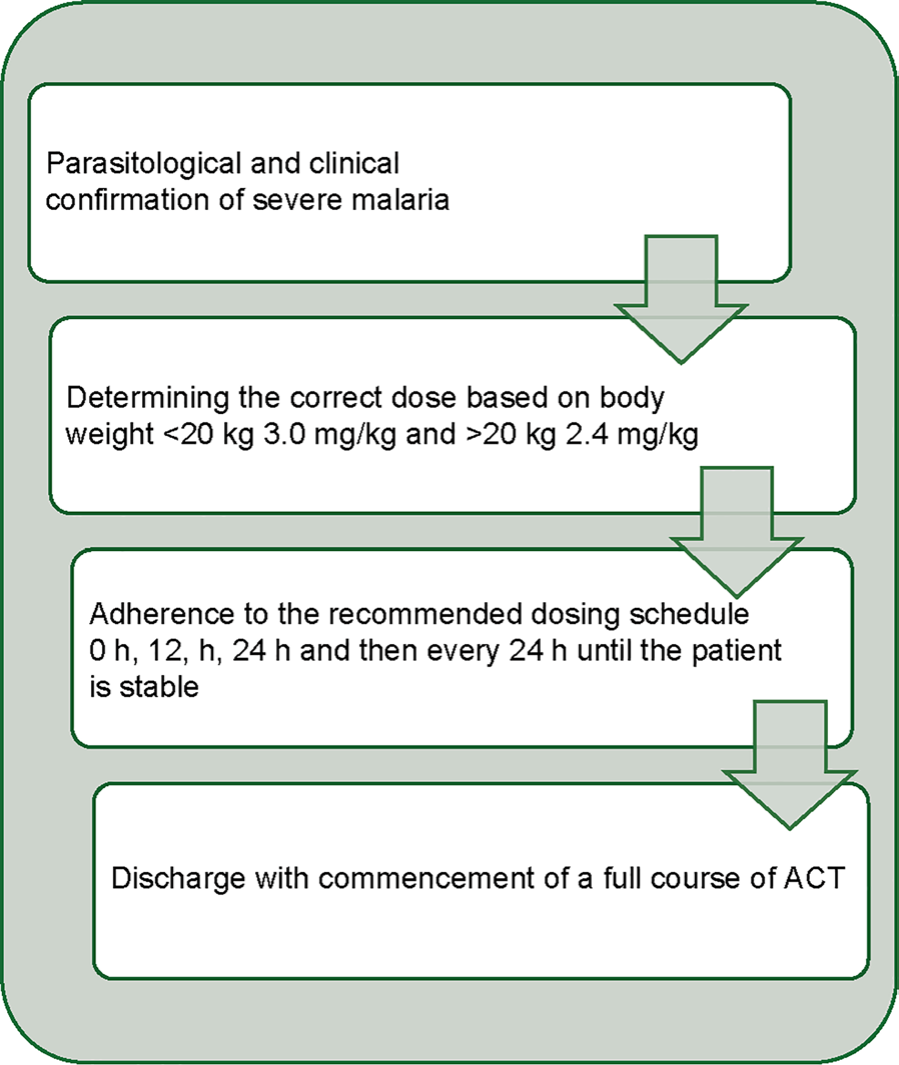
Appropriate administration of injectable artesunate

Mr John Isi, a representative of Tridem Pharma, highlighted the four key steps to ensure appropriate administration of injectable artesunate and follow-on with ACT in the treatment of severe malaria (Fig. 5). The appropriate reconstitution and dosing of artesunate powder is key to ensure that therapeutic doses are sufficient for clinical efficacy. This involves the addition of 5% sodium bicarbonate solution, mixing, and then the addition of 0.9% sodium chloride solution. The required amount of artesunate solution is drawn and the remainder is discarded. By deploying three strengths of injectable artesunate (30, 60, and 120 mg), dosing by patient weight is simplified and drug wastage minimized, with an average cost saving of 20% versus deployment of the 60 mg strength alone. Additional benefits include reduced transport and storage requirements and a reduction in healthcare providers' workload as the number of vials that need to be reconstituted is reduced. Thus, the deployment of three different artesunate strengths can contribute to improvements in logistics and capacity.

## Day 2 presentations

### Continuum of care for severe malaria

**Fig. 6:**
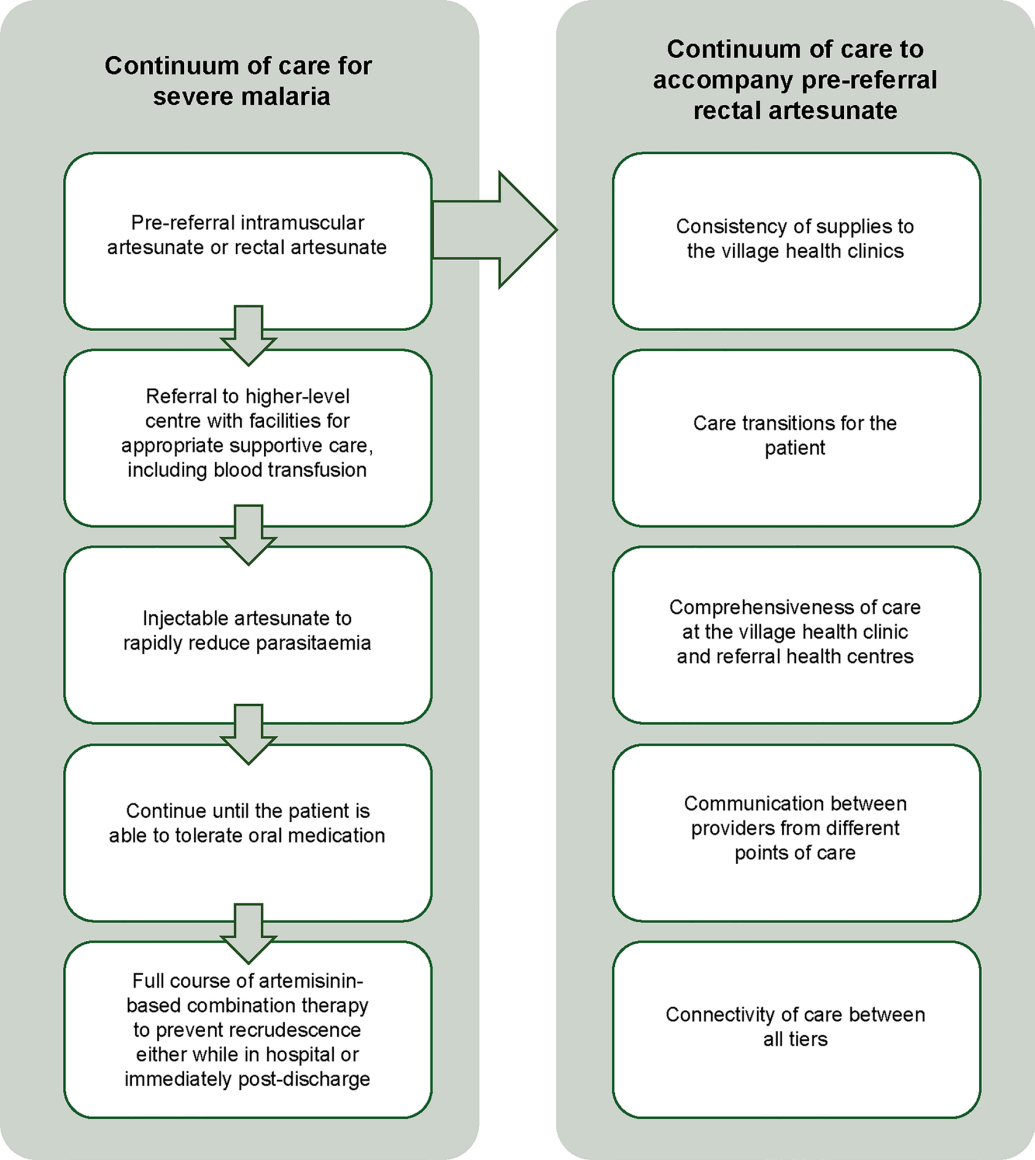
The continuum of care for severe malaria, and criteria to accompany rectal artesunate interventions [38]

The risk of death from severe malaria is around 50 times that of uncomplicated malaria. However, severe malaria does not get the attention that it warrants from international agents and policymakers, explained Hans Rietveld from MMV, Geneva, Switzerland. The continuum of care for severe malaria requires the availability of RAS in the community with urgent referral to higher-level health facilities for injectable artesunate followed by a complete course of ACT (Fig. 6). There are two WHO prequalified RAS preparations available and three injectable artesunate drugs. Recently, real-life stability data for rectal artesunate capsules from Nigeria’s Adamawa State has supported the WHO prequalification update to recommend a 6-month short-term stock of RAS in areas where the ambient temperature frequently exceed 30 °C. A new injectable artesunate product that only requires one-step reconstitution based on arginine rather than the current two-step process received WHO prequalification in 2023. This could further simplify the preparation of intramuscular and intravenous artesunate administration.

Although procurement of injectable artesunate increased between 2021 and 2022, sales of RAS have more than halved, in part because of an information note issued by WHO in 2022 which urged risk mitigation for the use of RAS over concerns for its effectiveness [39]. This was prompted by the preliminary results of observational studies from the Community Access to Rectal Artesunate for Malaria (CARAMAL) Project [40–43]. Clinical trials of RAS showed significant reductions in mortality and disability in children under 6 years of age who were referred to and reached a higher-level health facility more than 6 h after presentation [44]. As the window of opportunity to prevent death from severe malaria is short, RAS offered a solution in situations where referral was delayed. However, the initial review of CARAMAL by the WHO Global Malaria Programme and the Malaria Policy Advisory Group in 2021 indicated that the potential benefits of RAS did not translate into clinical effectiveness [39]. Subsequent scrutiny by a WHO technical consultation group led to an updated information note in July 2023 [45]. This highlighted the limitations of the CARAMAL study design. For example, in Nigeria the impact on the case fatality rate compared data from before RAS deployment and after RAS deployment, but there was temporal confounding with the evidence suggesting a lower underlying case fatality rate in the post-RAS period [45]. However, the study clearly identified the challenges in delivering the continuum of care for RAS and the deficiencies in health systems that must be addressed to ensure referral completion. Data from Malawi suggested five key criteria that are required for effective deployment of the intervention (Fig. 6) [38]. In Nigeria, the national treatment guidelines include RAS, and this intervention should be implemented, but with careful consideration of the supporting infrastructure and systems required to ensure effectiveness, particularly regarding referral completion.

### Severe malaria product quantification assumptions

The quantification of drugs and commodities for severe malaria case management is complex and challenging and depends on the availability of good quality data explained Sam Abutu from NMEP, Nigeria. In Nigeria, 100% of the population is at risk of malaria, but the population size must be projected based on 2006 census data adjusted for inter-census population growth. The average number of fever cases per year must be stratified by age as the annual incidence of fever decreases with age. The annual reduction in fever episodes and the percentage of fever cases seeking care must also be determined, in this case data from the Nigeria 2021 malaria indicator survey can be used. Assuming 2.2% of uncomplicated malaria cases progress to severe malaria and that 100% of these cases will get injectable artesunate, there will be 1.24 million severe malaria cases that receive artesunate in 2024. Given the average number of injectable artesunate vials per treatment is 3.125, 3.87 million injectable artesunate units are needed for 2024 in Nigeria.

## Roundtable discussion 1: effective management of severe malaria in Nigeria

**Fig. 7:**
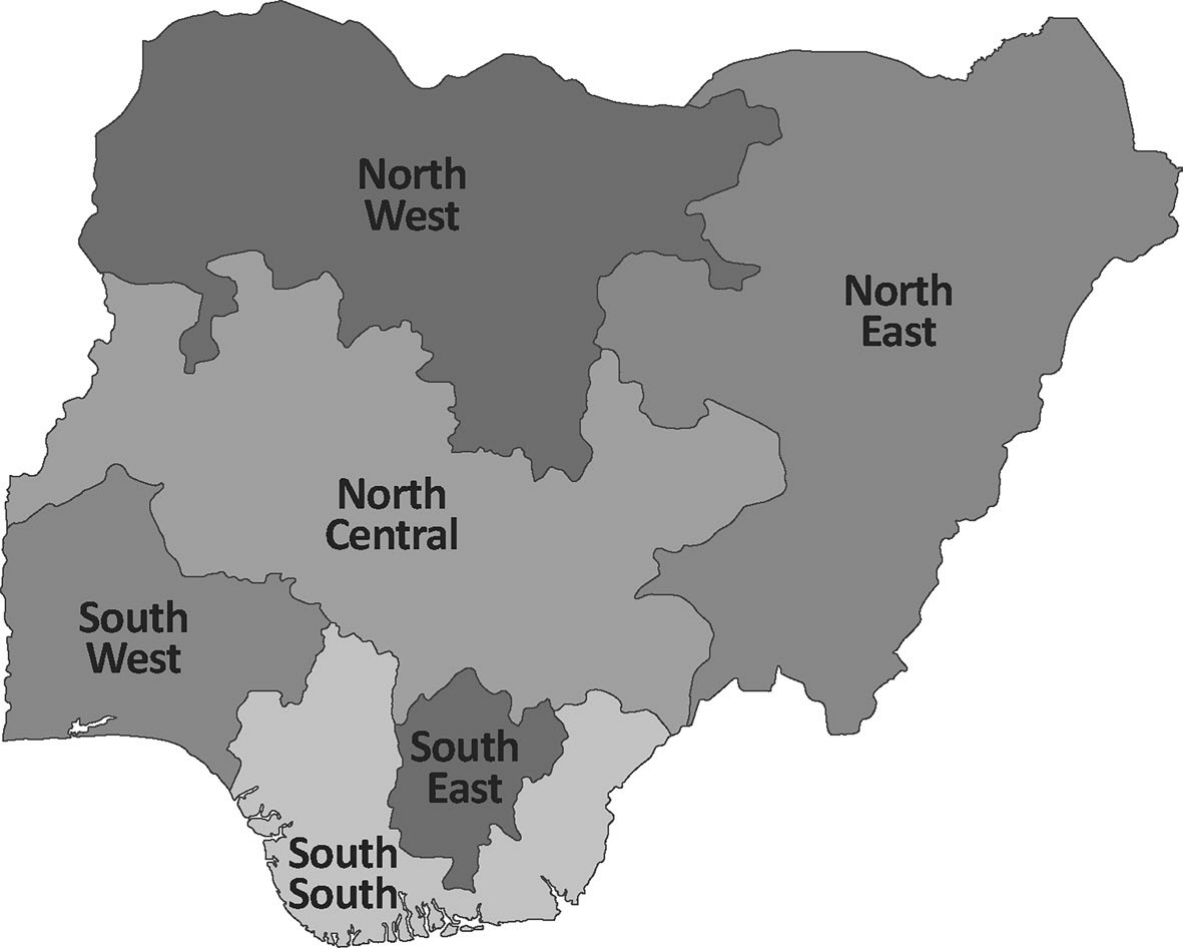
Geopolitical zones in Nigeria. North-Central: Benue, FCT, Kogi, Kwara, Nasarawa, Niger, Plateau. North-East: Adamawa, Bauchi, Borno, Gombe, Taraba, Yobe. North-West: Kaduna, Katsina, Kano, Kebbi, Sokoto, Jigawa, Zamfara. South-East: Abia, Anambra, Ebonyi, Enugu, Imo. South-South: Akwa-Ibom, Bayelsa, Cross-River, Delta, Edo, Rivers. South-West: Ekiti, Lagos, Ogun, Osun, Ondo, Oyo

**Fig. 8:**
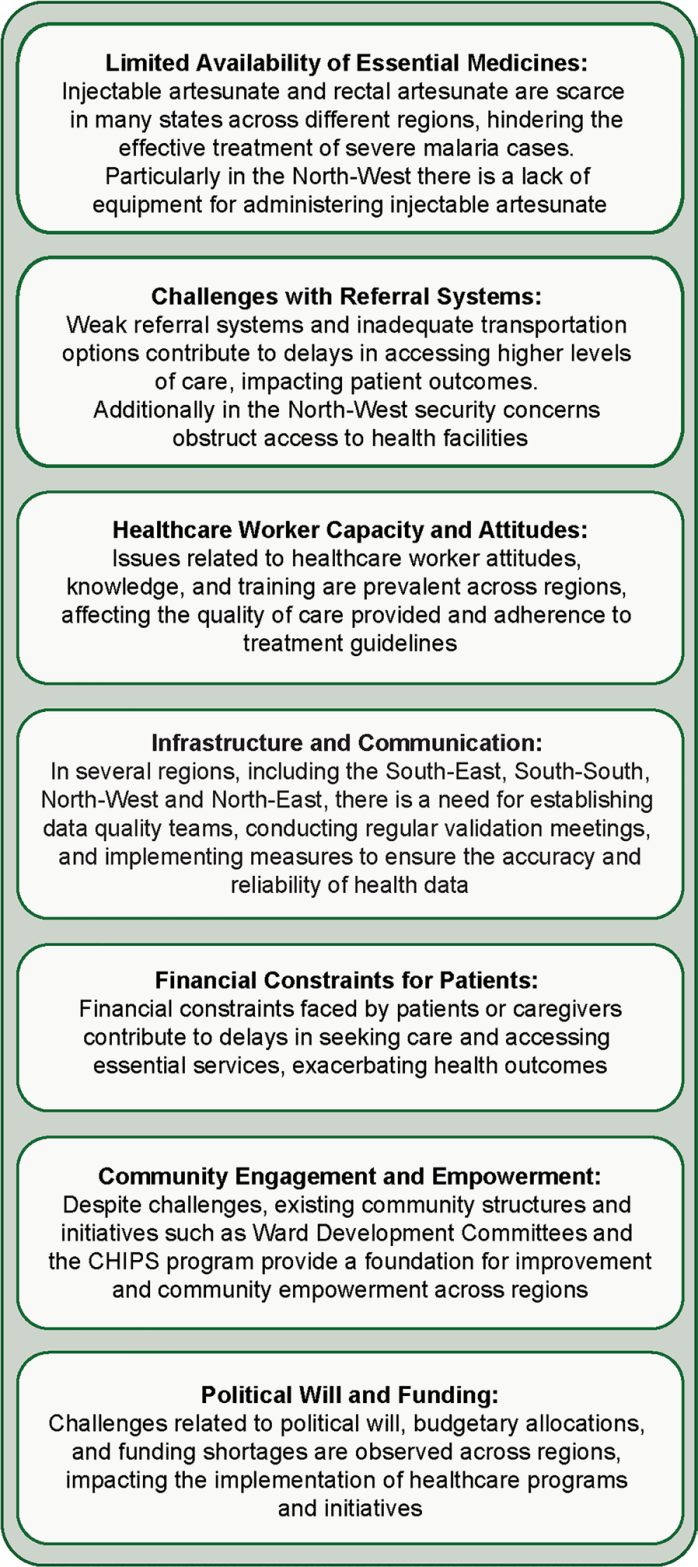
Cross-cutting themes in the effective management of severe malaria in Nigeria

Facilitated by Professor Olugbenga Mokuolu from the MSH, USA, participants were grouped by geopolitical zones (South-East, South-South, North-East, North-Central, North-West, and South-West) (Fig. 7) to discuss the challenges, facilitators and recommendations for the effective management of severe malaria. Cross-cutting themes are summarized in Fig. 8 and regional perspectives are outlined below.

In the South-East region, encompassing Abia, Anambra, Ebonyi, Enugu, and Imo states, injectable artesunate is available but expensive in some states, whereas RAS is scarce. Challenges include inadequate human resources, poor road infrastructure leading to high transportation costs, and a lack of political will to enhance the referral system. Additionally, suboptimal health-seeking behaviour at the community level and limited network connectivity, particularly in remote areas, exacerbate the situation. Despite these challenges, certain existing initiatives provide a foundation for improvement. Programmes like the CHIPS programme in some states and emergency transportation systems offer some support, as do well-functioning community structures such as the Ward Development Committee and financial schemes like the Mother Saving Loan Scheme in Ebonyi state. To address these issues, it is crucial to advocate to the State Primary Health Care Development Agency for the availability of essential commodities like RAS and injectable artesunate from the Basic Health Care Provision Fund. Additionally, implementing telehealth services would strengthen the referral system, while training key community stakeholders—including CHIPS agents, Ward Development Committee members, and emergency transport service drivers—would ensure they are adequately equipped. Furthermore, training community health workers on administering injectable artesunate and expanding ambulance and emergency transport systems are vital steps towards improving healthcare accessibility and outcomes in the region.

In the South-South region, including Akwa-Ibom, Bayelsa, Cross-River, Delta, Edo, and Rivers states, injectable artesunate is used when available and RAS is scarce. Challenges include poor attitudes among healthcare workers, a limited communication network, the absence of structured referral tools, financial constraints faced by patients or caregivers, and suboptimal emergency medical services. However, there are also positive factors such as the availability of standard operating procedures, structured referral tools, and behavioural change communication initiatives at the community level. Proposals included introducing innovative transportation solutions and implementing a two-way referral system to enhance communication channels. Additionally, training for health workers should be provided, a directory of referral centres to facilitate effective referrals must be established, and the community empowered to take charge of their healthcare needs.

In the South-West region, including Ekiti, Lagos, Ogun, Osun, Ondo, and Oyo states, injectable artesunate is available in some states at secondary and tertiary health facilities, but adherence to recommended dosing regimens is suboptimal. Challenges include the non-availability of referral forms, limited information and communication technology infrastructure hindering effective communication, and inadequate emergency medical services. Additionally, there are weak connections between different levels of care and a lack of structured two-way referral systems. However, some states in the region have implemented a hub and spoke model for referrals and offer centralized ambulance services. In remote areas, community tricycle ambulances are used at primary healthcare centres, with significant community involvement. A key recommendation for improvement was the enhancement of healthcare worker capacity through training and supportive supervisory visits. Also, stakeholder engagement is needed in advocacy efforts to boost political commitment. The widespread adoption of the hub and spoke system, improving the two-way referral process with necessary tools, and utilizing information communication technology, especially mobile phones, for better communication and feedback mechanisms were also recommended.

In the North-East region, encompassing Adamawa, Bauchi, Borno, Gombe, Taraba, and Yobe states, stock out of injectable artesunate is an issue and there were concerns regarding referral completion following RAS. Challenges include difficulties in reaching remote communities, weak connections between primary and secondary healthcare facilities, shortages in healthcare personnel, and issues with the attitudes of healthcare workers. Security concerns further compound these challenges, along with barriers stemming from poverty that hinder access to care. Addressing these issues requires strengthening facility linkages in the referral system and implementing regular monitoring and supportive supervision across all levels of care. Additionally, capacity-building initiatives for healthcare workers, the CHIPS programme, and for referral systems and algorithms should be prioritized. Improvements to emergency transport systems are also required, such as providing tricycles to healthcare facilities for improved access.

In the North-Central region, which includes Benue, FCT, Kogi, Kwara, Nasarawa, Niger, and Plateau states, injectable artesunate is widely used, but RAS is scarce. Challenges include poor documentation and insufficient pre-referral interventions, often due to the high cost of ambulances at health centres. Additionally, there is a lack of adequate referral tools and pre-referral drugs, along with a one-way referral system and shortcomings in the emergency transportation system. Limited funding and cultural barriers further impede healthcare access. However, there are existing community structures like the Women Development Committees that can be utilized to enhance the referral system at the community level, along with political will at the state level and support from international partners. Strategies to address these challenges involve expanding the reach of community structures such as CHIPS and Community Health Extension Workers responsible for referrals, promoting better collaboration, coordination, and leadership, and implementing evidence-based interventions to optimize limited resources. Participants underscored the importance of evidence-based systems to facilitate the physical transportation of patients across various healthcare levels.

The North-West region comprises Kaduna, Kano, Sokoto, Kebbi, Jigawa, and Zamfara states. While not all states in this area face the same challenges, many have reported issues such as the limited availability of injectable artesunate and RAS at primary healthcare centres. Health workers often resort to alternatives like intramuscular artemether or α-β-arteether due to their lack of skills for reconstituting injectable artesunate and a shortage of necessary basic equipment, like weighing scales. Consequently, sub-standard anti-malarials are commonly used to treat severe malaria cases. At the secondary and tertiary healthcare facility level, access to and usage of injectable artesunate is better, but stockouts of essential malaria commodities and sub-standard medications persist. The referral system operates poorly due to inadequate structure, topographic barriers, and in some areas security concerns, hindering access to healthcare facilities. Additionally, the absence of a functional two-way referral system and essential referral tools exacerbates operational challenges. Barriers to an effective referral system include socio-cultural factors, insecurity, limited understanding of malaria danger signs among caregivers, preference for alternative care, and chronic stockouts. Conversely, facilitators like health insurance schemes, emergency ambulance systems, and community resource persons are pivotal in managing uncomplicated malaria and preventing progression to severe malaria. Addressing these issues requires improving emergency ambulance coverage at the community level, expanding health insurance to the informal sector, and implementing RAS for pre-referral treatment at primary healthcare centres. Additionally, enhancing community awareness and advocacy on severe malaria and establishing linkages is essential.

## Roundtable discussion 2: severe malaria burden and data reporting from secondary and tertiary hospitals

Before the second roundtable discussion session, there were two short presentations to provide context and stimulate discussions relating to reporting and management of severe malaria data in Nigeria.

### Preparatory talk 1: Rapid impact assessment 2022

Cyril Ademu from the NMEP, Abuja, Nigeria, explained that the rapid impact assessment aimed to assess the effect of scale-up of malaria control interventions on the trend in malaria morbidity and mortality to determine the success of implementation and provide justification for resources mobilization between 2019 and 2021. Outpatient and inpatient malaria statistics were collected from selected health facilities for two age categories: under 5 years old and 5 years and older, spanning from 2019 to 2021. Additionally, data regarding State-level malaria intervention efforts from 2019 to 2021, along with the distribution of artemisinin-based combinations were collected and analysed. Overall, 1054 public and private tertiary and secondary health facilities were surveyed.

There were considerable regional differences in malaria incidence rates with seasonal variations in the North-West and Sahelian regions. The North-East had the highest burden of malaria out-patient cases, the highest burden of malaria outpatients who were children under 5 years old, the highest incidence of deaths from malaria, the highest incidence of malaria admissions and the highest all age malaria hospital mortality rate. However, the highest number and incidence of malaria inpatient admissions and deaths in children under 5 years old were reported in the North-East. Hospital mortality in children under 5 years of age was highest in South-South. Trends in the number of malaria or anaemia cases among outpatients or inpatients showed no significant differences between 2019 and 2021 overall. These findings provide key information on where activities and interventions can be best targeted in terms of addressing malaria burden, improving referrals, and enhancing the in-patient management of severe malaria.

### Preparatory talk 2: severe malaria reporting rates and burden

An analysis of data from District Health Information Software 2 (DHIS2) from January 2022 to May 2023 was presented by Cyril Ademu. Approximately 84% of states recorded a testing rate exceeding 85%, aligning with the National Malaria guideline of testing before treatment. However, Abia, Bayelsa, Enugu, Imo, Ondo, and Lagos States fell short of this threshold. Malaria test positivity rates varied, from 46% in Plateau state to approximately 94% in Abia State, with around 38% of states reporting rates below 60%. Few states treated severe malaria cases with injectable artesunate at rates exceeding 90%. Six states along with the FCT reported inconsistent data. Cross River state was the only state to report a high rate of oral artemisinin-based combinations follow-up for severe malaria cases, exceeding 90% from January to May 2023. Persistent data quality issues were noted, affecting service delivery in terms of commodity availability and patient management.

### Roundtable discussion 2: severe malaria burden and data reporting from secondary and tertiary hospitals

**Fig. 9:**
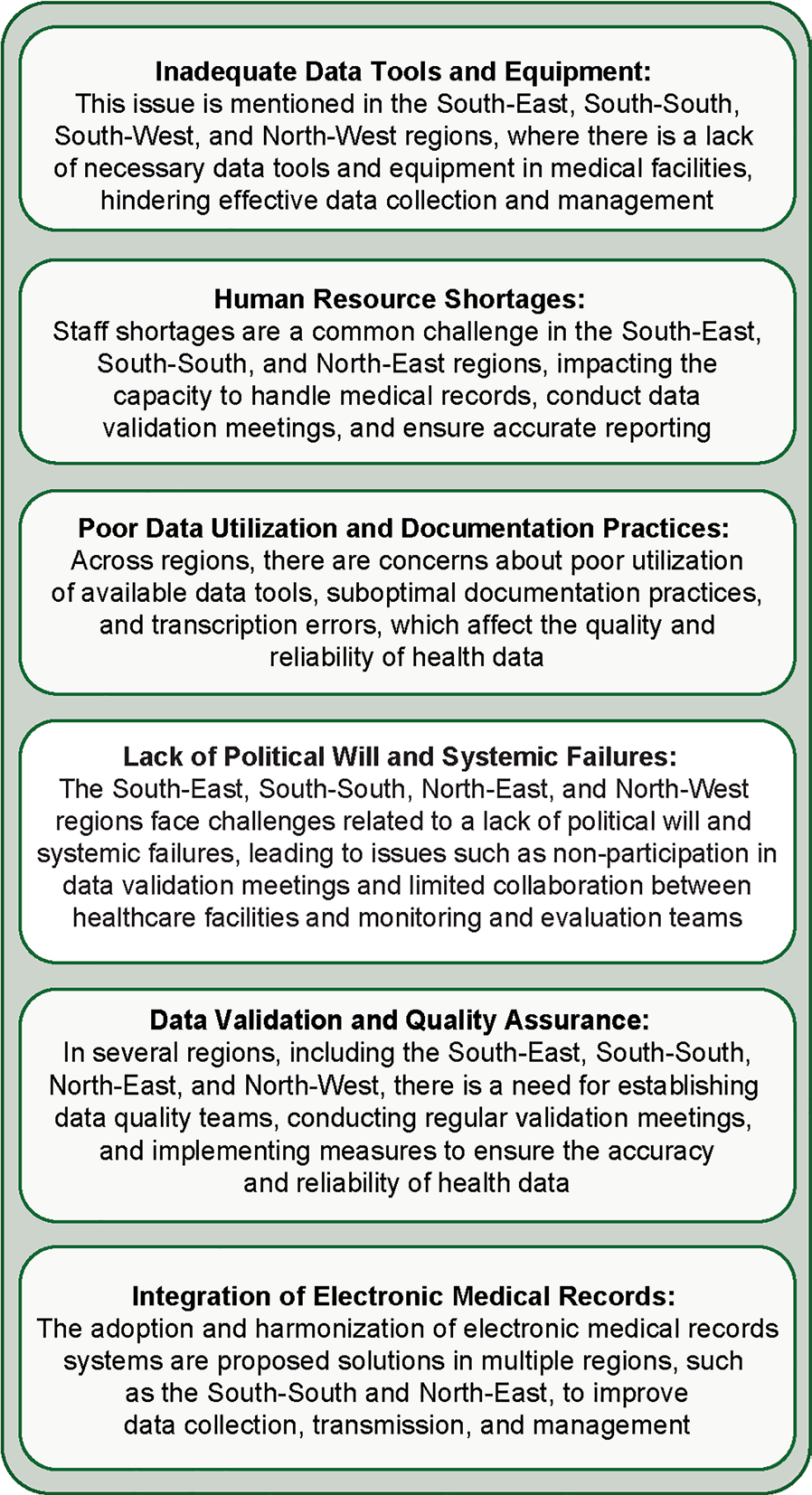
Cross-cutting themes in severe malaria burden and data reporting from secondary and tertiary hospitals

Participants were grouped according to the six geopolitical zones to brainstorm and discuss the burden of severe malaria data reporting from secondary and tertiary health facilities, identifying enablers, inhibitors, and recommendations for improved severe malaria reporting. Cross-cutting themes are outlined in Fig. 9, and challenges and recommendations by region are outlined below.

In the South-East region, challenges such as the non-availability of data tools in medical facilities, inadequate human resources in the medical records department, and a lack of utilization of available data tools persist. Additionally, there are difficulties in collecting reports from various service delivery points in hospitals, and secondary and tertiary facilities do not actively participate in monthly LGA data validation meetings due to factors like a lack of political will and systemic failures. To address these issues, interventions such as providing necessary data tools, recruiting and deploying trained personnel, ensuring political will, establishing data quality teams, and conducting training for record officers on electronic data capturing were recommended.

In the South-South region, there was poor clinician knowledge regarding severe malaria classification, inadequate adherence to testing guidelines, suboptimal documentation practices for a history of fever and diagnosis, poor completion of in-patient registers, and transcription errors in data registers. Furthermore, there was a lack of designated personnel for register completion due to staffing shortages and failure to compile data from all units of the tertiary health facility. In some areas data registers were not available. Proposed solutions involve training and retraining clinicians on severe malaria classification and reporting, training and retraining record officers on data quality management, raising awareness on guidelines, supplying data tools such as standard operating procedures and guidelines as well as data registers, increasing employment opportunities, and establishing data quality teams for monthly validation meetings.

In the South-West region, there was a high attrition rate of trained personnel, poor data capturing practices, insufficient equipment and data tools, and non-reporting of data to LGAs by healthcare facilities. To mitigate these challenges, recommendations included establishing data management teams, conducting monthly data validation meetings, providing visualization tools, encouraging clinicians to improve documentation practices, recruiting additional staff, promoting harmonization of electronic medical records with DHIS2, and fostering political support for data quality efforts.

In the North-East region, there were issues of staff shortages, incomplete documentation, excessive data reporting tools, poor data coordination, and limited collaboration between healthcare facilities and the LGA monitoring and evaluation teams. Proposed interventions included automating data collection processes by using electronic medical record systems, establishing data control rooms, and strengthening these at the LGA level.

In the North-Central region, challenges included poor record transmission from tertiary facilities to the LGA, inadequate coding mechanisms, lack of motivation, and disparities in disease coding. Solutions proposed involved harmonizing data systems in line with the National Health Management Information System (NHMIS).

In the North-West, reporting within secondary and tertiary health facilities faces several challenges: incomplete documentation, insufficient training of medical record officers on the NHMIS, and inadequate data tools. Coordination issues between various departments and poor linkages with LGA health authorities further complicate the data collection process. Despite these challenges, some facilities have implemented NHMIS data tools and established data quality review teams. To improve data reporting, recommendations include conducting routine data review meetings, establishing a malaria quality of care committee at each facility, continuous training of medical record officers, and implementing monthly data validation and triangulation to ensure accuracy and reliability of data.

## Conclusions

This workshop provided a forum to share updates on the management of severe malaria, identify challenges and barriers to effective care and identify potential solutions. The diversity of the participants enabled wide-ranging discussions and a practical and regionalized approach as well as providing an overview of severe malaria management in Nigeria.

Diverse challenges must be addressed if Nigeria is to reduce the burden of severe malaria and improve mortality rates. Frontline health workers lack adequate awareness of the revised national diagnosis and treatment guidelines for malaria, as well as other related strategic documents such as job aids and standard operating procedures. There is widespread misuse of non-recommended medicines, particularly α-β-arteether, for both severe and uncomplicated malaria treatment by frontline health workers. Wastage occurs during the reconstitution, dosage adjustment, and administration of artesunate injections with impacts on procurement and logistics as well as programmatic costs. Importantly, RAS, key for initiating pre-referral treatment, is unavailable in most states. Coordination and collaboration between different government agencies in developing strategic documents and guidelines are suboptimal. Referral linkages within the healthcare system require strengthening, and there are inadequate data management systems for documenting and reporting severe malaria cases in secondary and tertiary health facilities. Human resources for health are insufficient, with frequent staff attrition and non-recruitment by state governments. States also provide inadequate and irregular budgetary releases for implementing malaria programme activities. In response to these challenges, participants identified potential ways to enhance severe malaria management in Nigeria, outlined in the recommendations below.

### Recommendations

The meeting participants agreed on the following recommendations to improve the quality of care and data management for severe malaria in Nigeria:

1. To address the gap of poor awareness of frontline health workers of the existing updated guidelines and policy documents, the NMEP, SMEPs, and implementing partners are to employ innovative and effective channels of information dissemination. One such channel is the dissemination of information using professional associations which will, in turn, circulate within their network.
2. The need to adopt the newly released WHO information note on RAS (July 5, 2023) that reinforces the use of RAS for eligible age groups for pre-referral treatment.
3. Engagement with the National Primary Care Development Agency and relevant partners to strengthen the technical content and update the malaria component of the CHIPS training manual.
4. A consolidated call is made to all stakeholders for the discontinuation of the use of α-β-arteether injection for the management of uncomplicated or severe malaria by health workers in line with the diagnosis and treatment guidelines for malaria which recommend the use of ACT for the treatment of uncomplicated malaria and injectable artesunate for managing severe malaria with subsequent use of a full course of ACT when the patient can tolerate it orally.
5. Consistent with the updated WHO malaria guidelines [13], the recommended treatment for uncomplicated malaria during the first trimester of pregnancy is artemether-lumefantrine. Quinine, which was previously recommended, is now known to be associated with poorer pregnancy outcomes; it is no longer the recommended treatment in the first trimester of pregnancy. In the absence of artemether-lumefantrine, other artemisinin-based combinations can be used. However, there is currently no data on the use of artesunate-pyronaridine in the first trimester of pregnancy.
6. The introduction of multiple strengths of artesunate injection (30 mg, 60 mg, and 120 mg vials) offers opportunities to minimize wastage and reduce costs to patients. All unused volumes of reconstituted artesunate injection must be discarded after one hour of reconstitution, as by this time hydrolysis has degraded the drug and it cannot be administered to patients.
7. While newly available data (routine data in some states and research studies) suggest an increased prevalence of severe malaria in individuals > 5 years of age, it does not imply that children < 5 years old are no longer vulnerable to malaria and, therefore, all current recommendations regarding this age group remain applicable. There is however the added need for focused surveillance and strategies targeted at those at risk who are > 5 years old, including prioritizing commodities forecasting and procurement to cater to these older age groups.
8. A call for action on implementing the national guidelines on pre-referral treatment including making RAS widely available for use in children under 5 years.
9. The CHIPS programme provides an opportunity for improved access to quality case management of malaria and referral of severe cases from communities to PHC facilities. However, there is a need to strengthen referral systems to ensure the completion of treatment. Options for strengthening referrals include leveraging innovative collaborations with road transport union workers, emergency transport schemes, and the provision of relevant referral tools to improve referral at all levels of the health system. This includes deliberate actions for institutionalizing effective referral systems, such as special subsidy arrangements or public–private partnerships, which are backed by policy.
10. There is a need to scale up the use of electronic medical records and provide support in linking these with the DHIS2 platform to alleviate the burdens of data retrieval and reporting. This is also a call for novel strategies such as setting up data quality teams in secondary and tertiary health facilities to routinely collate and validate data generated within the health facilities for onward reporting.
11. Training and retraining of the medical records officers and service providers at the health facilities to strengthen data management and improve data quality.
12. States are encouraged to have annual recruitment and prompt replacement plans for health workers to sustain the quality of care for malaria.
13. Continuous advocacies by responsible Ministries Department Agencies to chief executives of states and FCT on the prompt release of allocated funds for the malaria programme implementation.

### Supplementary Information

Supplementary Material 1: Meeting agenda.

## Data Availability

All relevant data are provided in the manuscript or available from published materials as cited.

## Abbreviations

ACT: Artemisinin-based combination therapy
AQUAMAT: African Quinine Artesunate Malaria Trial
ASPIRE: Automated Software in the Identification of Malaria Retinopathy in Digital Retina Images of Cerebral Malaria
CARAMAL: Community Access to Rectal Artesunate for Malaria
CEFP: Community engagement focal persons
CHAI: Clinton Health Access Initiative
CHIPS: Community Health Influencers and Promoters Services
CRS: Catholic Relief Services
DHIS2: District Health Information Software 2
FCT: Federal Capital Territory
LGA: Local Government Area
MC: Malaria Consortium
MMV: Medicines for Malaria Venture
MSH: Management Sciences for Health
NMEP: National Malaria Elimination Programme
NHMIS: National Health Management Information System
PHC: Primary healthcare
PMI-S: US President’s Malaria Initiative for States
RAS: Rectal artesunate
SEAQUAMAT: Southeast Asian Quinine Artesunate Malaria Trial
SMEP: State Malaria Elimination Programme
SMWG: Severe Malaria Working Group
UNICEF: United Nations Children’s Fund
USAID: United States Agency for International Development
USAID IHP: United States Agency for International Development Integrated Health Programme
WHO: World Health Organization
