# Supplementary material for: Severe malaria intervention status in Nigeria: workshop meeting report
Source: Malar J. 2024 Jun 5;23:177. doi: 10.1186/s12936-024-05001-1 (PMC11155025; doi:10.1186/s12936-024-05001-1)
Supplement: Supplementary file 1 — Supplementary Material 1: Meeting agenda. [file 12936_2024_5001_MOESM1_ESM.docx]

**Additional file 1: Meeting agenda.**

| FEDERAL MINISTRY OF HEALTH | | | | |
| --- | --- | --- | --- | --- |
| NATIONAL MALARIA ELIMINATION PROGRAMME | | | | |
| ANNUAL SEVERE MALARIA STAKEHOLDERS MEETING | | | | |
| AGENDA | | | | |
| Date | | 5–6th July 2023 | | |
| Venue | | Sandralia Hotel, No 1, Solomon Lar Road, Jabi, FCT, Abuja | | |
| Time | | 8:30 am daily | | |
| Moderator | | Professor Olugbenga Mokuolu | | |
| Consultants | | Professors Oguche and JP Ambe | | |
| Meeting Objectives:  1. Provide an update and review progress on severe malaria activities in the country (NMEP and Partner’s projects), including the burden of the disease and commodity logistics management.  2. Disseminate recent research findings on severe malaria conducted globally and in-country and discuss implications for policy and implementation in Nigeria.  3. Provide an update and discuss ongoing efforts for routine data retrieval on severe malaria and ways to institutionalize this.  4. Define clear solutions and roadmaps to identified challenges with severe malaria management. | | | | |
| DAY 1 | | | | |
| S/N | ACTIVITY | | RESPONSIBLE | TIME |
| 1 | Arrival and registration of participants | | All | 8:30am – 9:00am |
| 2 | Opening prayer | | Volunteer | 9:00am – 9:05am |
| 3 | Self-introductions | | All | 9.05am – 9.15am |
| 4 | Meeting objectives | | H/CM | 9:15 – 9:20 am |
| 5 | Welcome remarks | | Director Public Health | 9:20am – 9:25am |
| 6 | Remarks | | Director Family Health  Director Hospital Services  Director Planning, Research, and Statistics  Director Disease Control NPHCDA | 9:25am – 9:45am    (3 MINS EACH) |
| 7 | Goodwill messages | | WHO, PMI, UNICEF, MMV, CHAI, MC, NMA, PAN, Rep of Teaching Hospitals | 9:45am – 10:05am    (2 MINS EACH) |
| 8 | Update on current Malaria National Guideline on Diagnosis and Treatment | | CM – NMEP | 10:05am – 10:35am |
| 9 | Overview of CHIPS: improving pre-referral and data management on severe malaria at community and PHC level | | NPHCDA | 10:35am – 10:50am |
| 10 | Q&As/discussions | | Facilitator | 10:50am – 11:15am |
| TEA BREAK | | | | 11:15am – 11:45am |
| 11 | Presentation on severe malaria retrospective studies (secondary and tertiary health facilities) | | Professor Oguche | 11:45am – 12:05pm |
| 12 | Presentation on death audit: what are the possible co-morbidities and pathological findings from postmortem investigation | | Tertiary health facility (Professor Ambe) | 12:05pm – 12:20pm |
| 13 | Q&As/discussions | | Facilitator | 12:20pm – 12:40pm |
| 14 | Presentation of QoC implementation in Kano state | | CHAI | 12:40pm – 1:00pm |
| 15 | PMI support for implementation of severe malaria intervention in Nigeria | | PMI-S | 1:00pm – 1:15pm |
| 16 | WHO presentation on antimicrobial resistance | | WHO | 1:15pm – 1:35pm |
| 17 | Q&As/discussions | | Facilitator | 1:35pm – 2:05pm |
| LUNCH | | | | 2:05pm – 3:00pm |
| 18 | Presentation on ASPIRE device | | Vision quest | 3:00pm – 3:20pm |
| 19 | Appropriate dosing of artesunate injection in optimizing treatment outcome for severe malaria | | Tridem Pharma | 3:20pm – 3:35pm |
| 20 | Questions and answers/discussions | | Facilitator | 3:35pm – 4:05pm |
| 21 | Wrap up and closing | | NMEP | 4:05pm – 4:30pm |
| Evening tea break | | | | |
| DAY 2 | | | | |
| S/N | ACTIVITY | | RESPONSIBLE | TIME |
| 1 | Recap of day 1 activities | | NMEP | 8:30am – 8:50am |
| 2 | Continuum of care for severe malaria: from community to tertiary care | | MMV | 8:50am – 9:20am |
| 3 | Severe malaria product quantification assumptions | | PSM - NMEP | 9:20am – 9:40am |
| 4 | Roundtable discussion: moderated by Professor Mokuolu  Effective management of severe malaria in Nigeria (utilization of injectable artesunate, RAS and referral system) | | Facilitators/NMEP/All. | 9:40am – 10:40am |
| TEA BREAK | | | | 10:40am – 11:10am |
| 5 | Presentation on 2022 rapid impact assessment | | M&E - NMEP | 11:10am – 11:20am |
| 6 | Presentation on severe malaria reporting rates and burden by states for secondary and tertiary healthcare facilities | | M&E - NMEP | 11:20am – 11:40am |
| 7 | Roundtable discussion (by zones):  Severe malaria burden and data reporting from secondary and tertiary hospitals: enablers, inhibitors and way forward for management of severe malaria | | Facilitators/NMEP/All | 11:40am – 12:40pm |
| 8 | Presentation from the 6 geopolitical zones:  (3 slides per zone) | | Presenters  (5 MINS EACH) | 12:40pm – 1:20pm |
| 9 | Discussions | | All | 1:20pm – 02:00pm |
| LUNCH | | | | 2:00pm – 3:00pm |
| 10 | Discussion: Support & commitment to improving severe malaria outcomes | | Facilitators/NMEP/All | 3:00pm – 3:40pm |
| 11 | Summary of key decisions and follow-up action points/communique | | Facilitators/communique drafting team | 3:40pm – 4:00pm |
| 12 | Closing remarks | | NC | 4:00pm – 4:15pm |
| 13 | Announcements and wrap up | | NMEP | 4:15pm – 4:30pm |
| EVENING TEA BREAK | | | | |
